# Susceptibility to Heat-Related Fluid and Electrolyte Imbalance Emergency Department Visits in Atlanta, Georgia, USA

**DOI:** 10.3390/ijerph13100982

**Published:** 2016-10-02

**Authors:** Leila Heidari, Andrea Winquist, Mitchel Klein, Cassandra O’Lenick, Andrew Grundstein, Stefanie Ebelt Sarnat

**Affiliations:** 1 Department of Environmental Health, Rollins School of Public Health, Emory University, 1518 Clifton Road NE, Atlanta, GA 30322, USA; lheidari@gmail.com (L.H.); awinqui@emory.edu (A.W.); mklein@emory.edu (M.K.); croleni@emory.edu (C.O.); 2 Department of Geography, Franklin College of Arts and Sciences, The University of Georgia, 210 Field Street, Athens, GA 30602, USA; andrewg@uga.edu

**Keywords:** climate change, temperature, heat morbidity, fluid and electrolyte imbalance, emergency department visits

## Abstract

Identification of populations susceptible to heat effects is critical for targeted prevention and more accurate risk assessment. Fluid and electrolyte imbalance (FEI) may provide an objective indicator of heat morbidity. Data on daily ambient temperature and FEI emergency department (ED) visits were collected in Atlanta, Georgia, USA during 1993–2012. Associations of warm-season same-day temperatures and FEI ED visits were estimated using Poisson generalized linear models. Analyses explored associations between FEI ED visits and various temperature metrics (maximum, minimum, average, and diurnal change in ambient temperature, apparent temperature, and heat index) modeled using linear, quadratic, and cubic terms to allow for non-linear associations. Effect modification by potential determinants of heat susceptibility (sex; race; comorbid congestive heart failure, kidney disease, and diabetes; and neighborhood poverty and education levels) was assessed via stratification. Higher warm-season ambient temperature was significantly associated with FEI ED visits, regardless of temperature metric used. Stratified analyses suggested heat-related risks for all populations, but particularly for males. This work highlights the utility of FEI as an indicator of heat morbidity, the health threat posed by warm-season temperatures, and the importance of considering susceptible populations in heat-health research.

## 1. Introduction

Climate change is predicted to increase the intensity, frequency, and duration of extreme heat events [1], particularly in large metropolitan areas [2]. Increased heat exposure has been identified by the World Health Organization (WHO) [3], the International Panel on Climate Change (IPCC) [4], and the US Global Change Research Program [5] as a major climate change-related health concern. Numerous studies have found a link between increased heat exposure and death [6,7,8,9]. Among the studies that have examined heat-related morbidity, investigators using emergency department (ED) visit and hospital admissions data have demonstrated an association between heat exposure and health care utilization for a variety of conditions including heat illnesses (defined as heat cramps, heat exhaustion, or heat stroke) [10,11,12,13,14,15,16,17], heart disease, renal disease, and diabetes [10,11,12,14,15,18,19,20]. Dehydration due to heat exposure may exacerbate these health conditions by increasing cardiovascular strain and impairing other thermoregulatory processes [21]; however, previous studies assessing susceptibility to heat have generally not considered dehydration-related health outcomes such as fluid and electrolyte imbalance (FEI). Furthermore, no studies to date have systematically assessed the influence of heat metric choice on the strength of association between FEI and heat; thus, FEI’s association with various heat metrics is not well characterized in the literature. Additional studies are needed to address this knowledge gap and identify the heat exposures that most influence FEI outcomes.

Despite being under explored, FEI morbidity has shown strong associations with high heat in Atlanta, Georgia [22] and California [10,11,12], and it has properties that make it a useful indicator of heat morbidity: (1) a diagnosis of FEI suggests general dehydration [23] and is made based on specific measurable conditions such as: hyperosmolality and/or hypernatremia, acidosis, alkalosis, mixed acid-base balance disorder, volume depletion (includes dehydration), fluid overload, hyperpotassemia, hypopotassemia, and other electrolyte and fluid disorders (e.g., electrolyte imbalance, hyperchloremia, and hypochloremia) [24]; and (2) the presence of FEI can be established relatively quickly and objectively through laboratory measurements combined with a physical examination and an assessment of the patient′s clinical history [25].

While there is some evidence that susceptibility to heat differs according to individual-level susceptibility factors, such as age [6,8,26,27,28], sex [6,26,27,29], race [8,9,30,31,32], as well as community-level factors, such as socioeconomic status [27,33], studies examining heat-related FEI morbidity among potentially sensitive subpopulations remains largely unexplored. Furthermore, susceptibility to heat may differ by presence of co-morbid/pre-existing health conditions and it is possible that such conditions may exacerbate a patient′s risk of dehydration with high temperatures. For example, some patient populations, such as those with congestive heart failure [34,35], myocardial infarctions [36,37], renal failure [38], and diabetes mellitus [39], are especially prone to FEI. Furthermore, medical visits for these types of conditions have been found to be associated with heat [8,11,12,15,22,40]. While FEI may be a pathway for increased susceptibility to heat for patients with these co-morbidities, to our knowledge, no previous studies have examined this question.

Additional studies examining heat-related morbidity are necessary for accurate climate-health risk assessments, and assessment of dehydration-related outcomes may improve estimations of the impact of heat on human health. Previous work in Atlanta found strong associations between warm-season same-day maximum temperature and maximum apparent temperature and FEI ED visits, with the strongest effects observed among patients in the 19–64 year old age group [22]. The present study expands on this analysis by estimating associations between heat and FEI morbidity among all ages for a variety of additional temperature exposure metrics (maximum, minimum, average, and diurnal change in ambient air temperature, apparent temperature, and heat index) and by examining heterogeneity of heat-morbidity associations by potential determinants of heat susceptibility (sex, race, comorbid congestive heart failure, kidney disease, and diabetes diagnoses, and neighborhood socioeconomic conditions).

## 2. Materials and Methods

### 2.1. Emergency Department Visit Data

Emergency department visit data for 20-county Atlanta, Georgia spanning the period 1 January 1993 to 31 December 2012 were previously collected for the Studies of Particles and Health in Atlanta [22,41,42,43,44,45,46]. Briefly, data on ED visits to hospitals in the Atlanta metropolitan area were obtained from individual hospitals (for the period 1993–2004) and from the Georgia Hospital Association (for the period 2005–2012). Data elements of specific interest to this analysis were admission date, primary and secondary International Classification of Diseases 9th Revision (ICD-9) diagnosis codes, patient age, sex, race, and ZIP code of residence. Data were restricted to patients whose residential Zone Improvement Plan (ZIP) code was at least partially within one of the 20 counties in the Atlanta metropolitan area. Use of the emergency department data was in accordance with agreements with the hospitals and the Georgia Hospital Association, and this study was approved prior to its conduct by the Emory University Institutional Review Board.

ED visits for FEI among all ages were identified as visits with an ICD-9 diagnosis code of 276. To optimize specificity of disease classification we only included ED visits with a primary diagnosis of FEI. These data were then aggregated to daily counts of ED visits for FEI overall, and for strata based on patient sex (female/male), patient race [nonwhite (black/Hispanic/other)/white], the presence (yes/no) of comorbidities including congestive heart failure (ICD-9 code 428), kidney disease (ICD-9 codes 580-593), and/or diabetes (ICD-9 code 250 or 249 on or after 1 October 2008). Comorbidities were identified via the presence of ICD-9 codes for these conditions in secondary diagnosis variables for the same visit. 

Daily FEI ED visit counts were also stratified by area-level socioeconomic indicators of poverty and education using ZIP code tabulation area (ZCTA; Census Bureau boundaries created from census blocks to approximate ZIP codes) information from the 1990 and 2000 US Census long form and the American Community Survey (ACS) five-year (2007–2011) summary file, all normalized to 2010 ZCTA borders [47]. Data from the different data sources were used to account for possible changes in area-level socioeconomic status (SES) over the 1993–2012 study period. We assumed that any changes occurred gradually, and thus assigned values of each SES variable to each ZCTA by year, weighted by proximity in time to the data source [48]. These data were merged with ZIP code level ED data by year. Ultimately, daily counts of ED visits for FEI were aggregated by “poverty area” defined as ZCTAs with ≥20% of the population living below the federal poverty line (yes/no), and “undereducated area” defined as ZCTAs with ≥25% of the adult population without a high school graduation (yes/no).

### 2.2. Meteorological Data

Meteorological data, including daily metrics (maximum, minimum, average, and diurnal change) for ambient air temperature and dew point temperature, were obtained from the Automated Surface Observing Station (ASOS) located at Atlanta Hartsfield International Airport for the 1 January 1993 to 31 December 2012 period. We also considered apparent temperature and the heat index, a variation of the apparent temperature used by the National Weather Service [49,50]. These indices combine the influences of air temperature and humidity.

### 2.3. Epidemiologic Analysis

Analyses for the current study were restricted to the warm season, as defined by the period from May to September. Associations between warm-season temperatures and FEI ED visits were estimated using Poisson generalized linear models allowing for overdispersion. We assessed and compared associations for various temperature metrics, including: maximum, minimum, and average ambient air temperature; maximum, minimum, and average apparent temperature; maximum and minimum heat index; and diurnal change in ambient temperature and apparent temperature. Analyses were conducted for each strata of interest to examine the heterogeneity of effects by potential determinants of heat susceptibility with daily maximum temperature (TMax) and daily maximum apparent temperature (ATMax). In all analyses, temperature metrics were modeled using linear, quadratic, and cubic terms to allow for possible nonlinear relationships with FEI ED counts [22]. All analyses considered same day (lag 0) temperatures, as this lag showed the strongest associations with FEI in our previous work [22]. Covariates in the model were consistent with those used in our previous work and included: control for time using a cubic spline with monthly knots within the warm season for each year; indicator variables for year, and terms for the interaction between the linear term for day of the warm season and each year indicator variable; indicator variables for day of week, federal holidays, and periods of hospital participation; and same day average dew point temperature modeled using cubic terms (except for models of apparent temperature and heat index metrics, as these include dew point information). All analyses were also conducted with data restricted to the time period 1998–2012, as low mean daily ED visit numbers in earlier years led to model convergence issues for some strata of interest.

We used the approximate interquartile range (IQR; 25th–75th percentile) of the temperature metric of interest to represent a meaningful increment in exposure for expressing rate ratios (RR). The 25th percentile was used as a reference temperature in estimating RRs for other temperature increments. We calculated these estimates by applying the parameter estimate values from the linear, squared, and cubic terms for the temperature metric of interest to the specific temperature contrast being considered [22].

Associations across subgroups (e.g., of sex, or of race) were compared as an assessment of heterogeneity of the overall heat-morbidity relationship. Significant differences in the rate ratio for the interquartile range for each strata of each heat vulnerability determinant were tested using a Wald test for heterogeneity [51]. All analyses were performed using SAS 9.4 (SAS Institute, Cary, NC, USA).

## 3. Results

### 3.1. Data Summary

During 1993–2012 in Atlanta, our database captured 66,369 ED visits for FEI that were made during the warm seasons (May–September) (*n* = 3060 days, mean of 21.7 visits/day). Table 1 shows descriptive statistics of the various temperature metrics for the study period. Table 2 shows that the maximum, minimum, and average temperature, apparent temperature, and heat index metrics were moderately to highly correlated with one another (Spearman correlation coefficients > 0.70). The diurnal change in temperature (TDC) and apparent temperature (ATDC) metrics were highly correlated with each other (Spearman correlation coefficient = 0.96) and showed weaker correlations with the other temperature metrics. Specifically, TDC and ATDC were negatively correlated with minimum temperature metrics (correlations ranging from −0.19 to −0.30) and were positively correlated with maximum temperature metrics (correlations ranging from 0.20 to 0.43), suggesting that within-day changes in temperature were smaller with increasing temperature lows and larger with increasing temperature highs.

### 3.2. Associations of FEI ED Visits and Warm-Season Temperature

The estimated rate ratios for all FEI ED visits for a 1 IQR change in temperature (from the 25th to the 75th percentile) during 1993–2012 were positive and statistically significant for all temperature metrics (Table 3). The strongest RR was for average apparent temperature (ATAvg) with an RR of 1.137 [95% confidence interval (CI): 1.113, 1.162] per IQR change from the 25th to 75th percentile. The weakest RRs were for minimum apparent temperature (ATmin), minimum heat index (HIMin), and the two diurnal change metrics (RRs ≤ 1.096 per IQR change from the 25th to 75th percentile); 95% confidence intervals were generally non-overlapping with those for the other metrics suggesting significantly weaker estimated effects. The RRs for maximum and average temperature and apparent temperature metrics were very similar, likely due to their high correlation (Table 2). Observed associations for TMax [RR of 1.125 (95% CI: 1.102, 1.150) per IQR] and ATMax [RR of 1.125 (95% CI: 1.103, 1.149)] were consistent with results presented in Winquist et al. (2016) [22].

**Figure 1:**
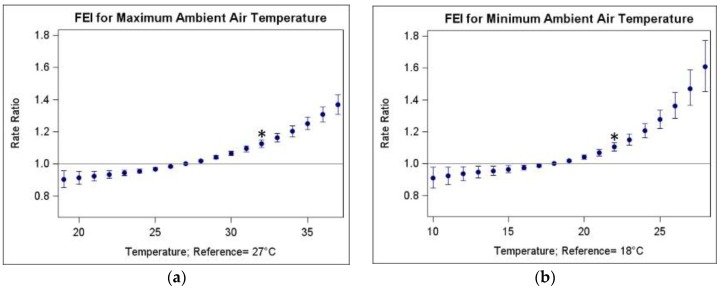

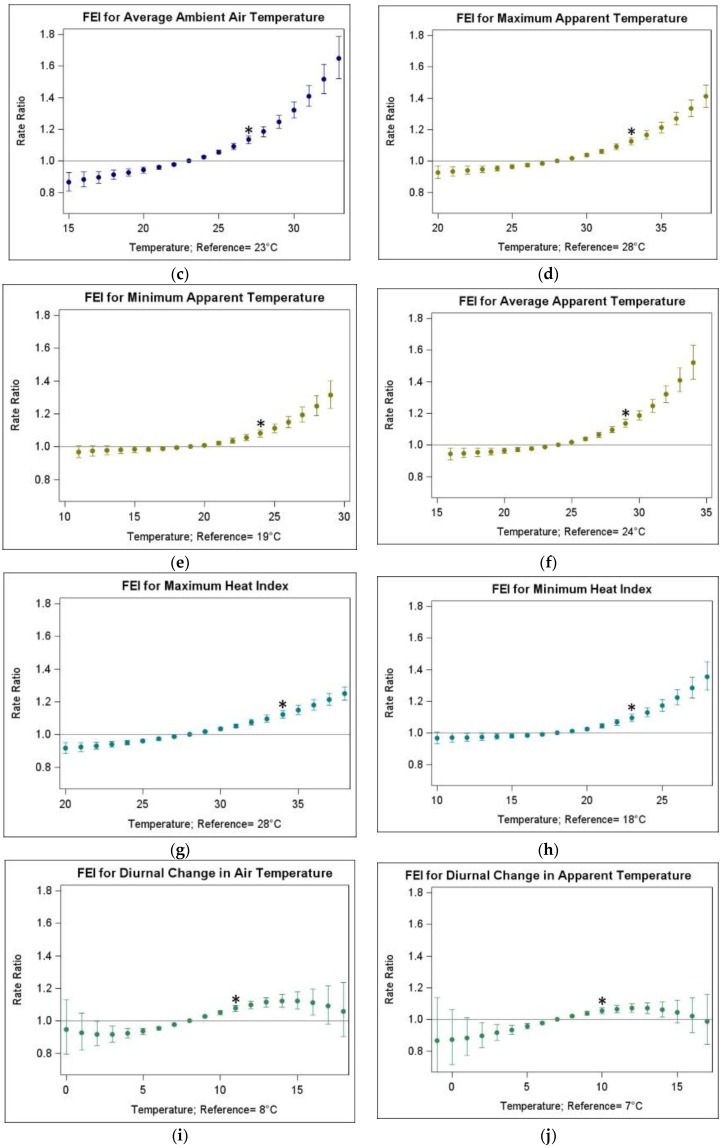
Plots of estimated rate ratios for fluid and electrolyte imbalance ED visits in relation to various temperature increments from the 25th percentile reference for various temperature metrics in Atlanta during warm seasons (May–September), 1993–2012: (**a**) Maximum ambient air temperature; (**b**) Minimum ambient air temperature; (**c**) Average ambient air temperature; (**d**) Maximum apparent temperature; (**e**) Minimum apparent temperature; (**f**) Average apparent temperature; (**g**) Maximum heat index; (**h**) Minimum heat index; (**i**) Diurnal change in air temperature; (**j**) Diurnal change in apparent temperature. * RRs marked by asterisk are equivalent to those reported in Table 3 and represent the RR per IQR from the 25th to 75th percentile.

Figure 1 shows estimated RRs across different temperature increments for each temperature metric, using the 25th percentile of each metric as the reference. For most metrics, we observed increasing RRs with increasing temperature compared to the 25th percentile reference temperature. TDC and ATDC metrics that had the weakest RRs per IQR (Table 3), also showed the widest 95% CIs and most curvilinear trends, with RRs decreasing at upper ranges (i.e., at larger ranges of diurnal temperature change). Given positive correlations of TDC and ATDC with maximum temperature metrics (Table 2), these trends may be due to days that have high maximum and low minimum temperatures, which allow people to cool off at night and may mitigate heat illnesses.

### 3.3. Heterogeneity of Heat Morbidity Associations

Further analyses were conducted to examine the heterogeneity of heat effects by potential determinants of heat susceptibility. Table 4 shows descriptive statistics of FEI ED visits stratified by all potential modifiers of interest in Atlanta during the warm seasons for 1993–2012. Overall, there were more visits by female compared to male patients, by white compared to nonwhite patients, by patients without comorbid than with comorbid conditions, and by patients from non-poverty and non-undereducated areas compared to other areas. Modifier information was missing for some ED visits, particularly for race for which 33% (*n* = 21,985) of visits were missing race information. The missingness in the race data was in part due to three years (2007–2009) for which information on patient race was completely missing from our database.

Analyses by strata of potential determinants of heat susceptibility focused on the TMax and ATMax temperature exposure metrics. Table 5 presents estimated rate ratios for FEI ED visits in relation to TMax and ATMax changes from the 25th to 75th percentiles, both overall and by strata of each potential heat susceptibility determinant. Models were run for both full (1993–2012) and reduced (1998–2012) time periods as low mean daily ED visit numbers in earlier years led to model convergence issues for some strata of interest. Among stratified analyses, most strata showed significant positive associations regardless of temperature metric; results were also consistent across the two time periods for strata without convergence problems. Statistically heterogeneous estimated effects were observed between categories of sex, with RRs for males [e.g., RR of 1.176 (95% CI: 1.138, 1.216) per IQR TMax during 1993–2012] significantly stronger than for females [e.g., RR of 1.088 (95% CI: 1.060, 1.118) per IQR TMax], and categories of renal disease comorbidity, with RRs among patients without a comorbid renal disease diagnosis {e.g., RR of 1.137 (95% CI: 1.112, 1.164 per IQR TMax} during 1993–2012 significantly stronger than RRs among patients with a comorbid renal disease diagnosis {e.g., RR of 1.048 95% CI: (0.995, 1.104) per IQR TMax}. Some suggested differences in estimated effects were found for race, with RRs among Hispanics considerably stronger than those for other groups, and undereducated area, with stronger RRs among patients residing in an undereducated area than those residing in other areas; however, these differences were not significant at the 0.05 level.

**Figure 2:**
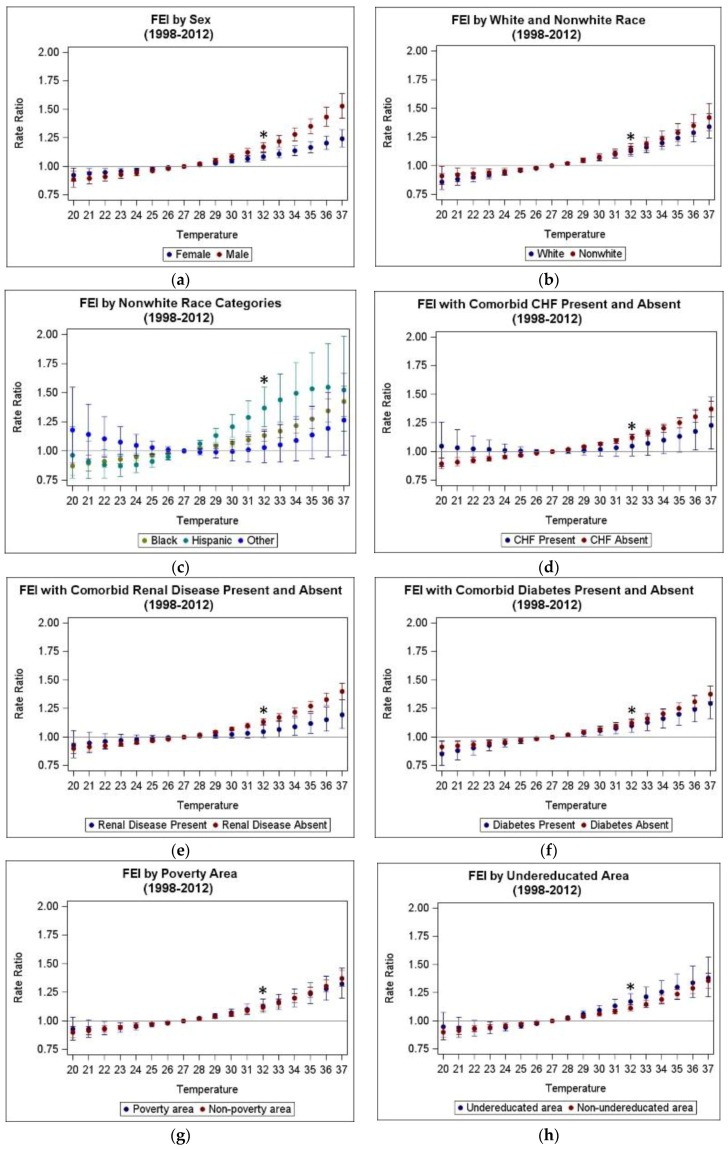
Plots of estimated rate ratios for fluid and electrolyte imbalance ED visits in relation to various temperature increments from the 25th percentile reference for TMax by modifiers of interest in Atlanta during warm seasons (May–September), 1998–2012: (**a**) FEI by Sex; (**b**) FEI by White and Nonwhite Race; (**c**) FEI by Nonwhite Race Categories; (**d**) FEI with Comorbid Congestive Heart Failure (CHF) Present and Absent; (**e**) FEI with Comorbid Renal Disease Present and Absent; (**f**) FEI with Comorbid Diabetes Present and Absent; (**g**) FEI by Poverty Area; (**h**) FEI by Undereducated Area. * RRs marked by asterisk are equivalent to those reported in Table 5 and represent the RR per IQR from the 25th to 75th percentile.

Figure 2 shows stratum-specific RR estimates expressed for a range of TMax increments, using 27 °C (25th percentile) as a reference, for the reduced time period (1998–2012). These plots show that the RR per IQR estimates presented in Table 5 adequately represent effect modification for each factor, and that the differences in RRs observed for sex and renal disease persisted at the upper temperature increments and were not unique to the RR per IQR change from the 25th to the 75th percentile. While most strata showed trends that were somewhat linear across the range of temperature increments, curvilinear trends appeared for nonwhite strata of Hispanic and Other.

## 4. Discussion

Using a 20-year time series of ED visits and ambient temperature in Atlanta, Georgia, this study evaluated heat-morbidity associations for FEI by potential determinants of heat susceptibility (sex, race, comorbid congestive heart failure, kidney disease, and diabetes diagnoses, and neighborhood socioeconomic conditions as defined by poverty and education levels), and explored the effects of defining exposure differently with a variety of temperature metrics. Overall, we found that warm-season same-day temperature is associated with FEI, regardless of the metric used to define temperature. Our results suggest that risks are great for all populations, and particularly great for males. We found some unanticipated patterns of effect modification for the comorbidities, as there was a tendency for the “absent” strata to have stronger associations than “present” strata, with significant differences observed for comorbid renal disease. Below we explore the implications of our observed results.

Previous studies have shown mixed evidence for modification of heat-health associations by sex. A number of studies have shown stronger heat-related morbidity for females [52,53,54,55,56,57,58,59,60,61,62,63], but other studies have found stronger effects for males [26,29,64,65] as was found in our study. The finding of males having a significantly stronger RR than females is supported by studies of ED visits [66] and hospital admissions [64] that have shown that men are more likely than women to seek care for heat-related diagnoses [20]. Males may be more likely to be exposed than females, based on occupational or recreational activities [64,66]. It is possible that differing physiological and metabolic processes between males and females also play a role; for example, males have a higher metabolic rate, related to higher muscle mass and lower subcutaneous adipose tissue, compared to females [66]. This greater burden of metabolic heat gain, when combined with heat exposure, may impair males′ abilities to disperse heat and lower their core body temperature. In our study, we assessed effect modification by sex across a broad age range, similar to other heat-health studies; yet susceptibility due to male or female sex may change as people age, which future research might further explore.

We observed significant positive associations for all race categories (except “other”). Stronger associations were generally observed among nonwhite patients than white patients, although these differences were small (i.e., for TMax during 1993–2012, RR among nonwhite patients was ~1% different from white patients); Hispanic patients appeared to drive the elevated RR for nonwhite patients (i.e., for TMax during 1993–2012, RR among Hispanic patients was ~20% different from white patients). While Hispanic patients appeared to have considerably stronger risks than other racial categories, this may be a chance finding; there were considerably fewer cases in this category (1938 ED visits; 3% of visits for race strata) compared to other race strata and missingness among our race data was high. However, these results are suggestive and warrant further investigation; in previous studies, Hispanic ethnicity has been shown to be associated with heat-related health impacts [14,67]. It is also important to consider that race may function as a distal measure of susceptibility [68] and that it may be more relevant to study race in conjunction with other measures of potential susceptibility which have been shown to be associated with being a racial/ethnic minority, such as income, health status, built environment characteristics related to heat exposure, air conditioning use, and occupational heat exposure [32,69,70,71].

We examined two area-level susceptibility measures in this study: neighborhood poverty and education. In stratified analyses, RRs were positive and significant across all strata indicating that heat exposure influences FEI in our patient population regardless of neighborhood poverty or education levels. While no differences in RRs were found between patients living in poverty and non-poverty areas, there was an indication, albeit not statistically significant, of stronger associations for patients living in undereducated compared to educated areas. Low ED visit counts within undereducated stratum and wide confidence intervals around RRs contributed to lack of detection of statistical differences between undereducated and educated strata. In the literature, there is mixed evidence regarding education and heat mortality and morbidity [8,9,30,68,72,73,74]. It is important to consider that single measures of socioeconomic composition are poor proxies for nuanced neighborhood environments and education levels may be associated with other facets of socioeconomic conditions in neighborhoods, including income level, mix of occupations that influence heat exposure (e.g., agricultural workers), and awareness of health risk [68] that complicate direct interpretation of results across studies. Furthermore it may be a challenge for certain populations to access appropriate fluids and in sufficient quantity to combat FEI. Given the many influences on heat exposure, individual-level circumstances (e.g., occupation, adequate housing, access to air conditioning) may be more important than neighborhood contexts with regard to modification of heat-FEI associations.

In this study, we focused our analyses on the warm season, with the consideration that the potential health threat posed by increased ambient air temperatures is likely to occur during periods of warm weather. It is important to note that this time of year is also a time during which populations are primed for adaptive behaviors to protect against heat morbidity and mortality such as using air conditioning, staying inside, and drinking more water. Hence, overall observed associations in our study naturally account for adaptive behaviors of the population overall during warm weather. While it was not possible to address here, it is also likely that certain subpopulations are more responsive to ambient heat during the warm season than the overall population due to individual behaviors or lack of access to resources that impact their level of personal exposure to heat and ability to cope with such exposure [32,69,70,71].

For the comorbidities of interest, there was a tendency for the “absent” strata to have stronger associations than “present” strata. This may be due to a number of interrelated reasons. First, it is important to consider that observed effect modification depends on whether one is considering multiplicative effects (as in this study) or additive effects, and that there may be complex and competing risk factors at play in these relationships. For populations with multiple risk factors for the outcome, which may be the case with patients with underlying chronic diseases, the risk due to temperature may be small relative to the other risk factors; thus, in multiplicative models, it is possible for the population with more risk factors to have smaller RRs than that for the population with fewer risk factors, which is the pattern observed in the current study. It may also be the case that people with underlying conditions are more aware of heat-related risks and take more behavioral measures to limit their heat exposures.

However, it is also important to consider the definition of comorbidity used. The present study′s definition of comorbidity aimed to capture individuals who presented as a new case of FEI, but who had an underlying condition represented in a secondary diagnosis code at the same visit at which FEI was coded as the primary diagnosis. This definition was intended to capture patients with an underlying disease, but for which manifestations of that condition were not severe enough for it to be coded as the primary diagnosis. Other studies of heat morbidity using ED visits have found significant associations with high temperatures for the comorbidities of interest (renal disease/failure, diabetes, cardiovascular diseases/outcomes other than congestive heart failure) [11,22,75]. It is possible that some patients in those studies also experienced FEI, but FEI was not coded as the primary diagnosis. Thus, similar to the proposed explanation that cardiovascular morbidity cases may be missed because they progress too quickly to mortality [22,76,77], it is possible that FEI cases progress quickly to a more severe manifestation of an underlying disease, and are therefore not coded as primary FEI.

The close relationship between fluid and electrolyte imbalance and kidney function may also make it challenging to disentangle the relative associations of the two with heat. Fluid and electrolyte imbalance affects renal function, and renal function affects how well fluids and electrolytes are balanced in the body. For example, as renal function declines, the kidneys cannot maintain homeostasis, leading to potential hyponatremia and hypernatremia, which are types of FEI [78]. The selected comorbidities for the current study are all linked to renal function. Diabetes can lead to chronic renal failure/impairment [79], and congestive heart failure patients often take medications that can impact renal function [34].

Studies have used a variety of different temperature metrics to assess heat exposure, including ambient air temperature and measures that integrate the influence of temperature and humidity, such as apparent temperature and the heat index. Different daily measures, expressed as maximum, minimum, or average measures of these temperature metrics, may indicate different mechanisms and effects. For example, heat-trapping urban environments may intensify heat stress by preventing overnight cooling, which is indicated by minimum air temperature [58,80]. Average temperature most likely represents the experience of the entire day and night [76,81]. ED visits for cardiovascular and respiratory diseases have been shown to be positively associated with diurnal temperature range when it was above a certain range [82,83]. Because each measure of temperature may be suited for certain scenarios and because FEI has not been previously studied in depth as an outcome in relation to heat exposure, an objective of the present study was to characterize the heat-FEI morbidity relationship for different exposure metrics.

Previous studies of temperature metrics in relation to FEI morbidity have either considered “dehydration”, identified by the specific ICD-9 code 276.5 [11,12], or have considered the full group of FEI ICD-9 codes (276) or ICD-10 codes [10,15,80,84] as done here. Our overall results are consistent with these studies, which found apparent temperature to be positively associated with ED visits for dehydration [11] and hospital admissions for dehydration [12], and also found positive associations for FEI emergency hospital visits on days of extreme heat [80] and for FEI ED visits and hospitalizations during a heat wave period [10]. In our analyses, all of the temperature metrics considered yielded statistically significant positive RRs for all FEI ED visits (between 1.055 and 1.137). While our outcome was defined to maximize specificity for FEI (i.e., by including only ED visits with a primary diagnosis of FEI), we note that by not including secondary diagnoses of FEI or a broader suite of symptoms related to heat stress (e.g., faintness) our results may be underestimates of the total effect of heat on FEI and related symptoms. When comparing RRs across different temperature metrics, we found that measures of apparent temperature and heat index, both of which include measures of humidity in their calculation, yielded similar RRs, and that RRs for apparent temperature were similar to corresponding measures of temperature (i.e., TMax vs. ATMax, TMin vs. ATMin, TAvg vs. ATAvg). Measures of diurnal temperature change (TDC, ATDC) yielded weaker RRs than the other metrics, indicating that this may not be the most important heat-related risk factor for FEI. All of these temperature metrics were highly correlated with one another, except with TDC and ATDC. Our results suggest that temperature, apparent temperature, and heat index may be regarded as similar risk factors for dehydration-related health effects; this finding is relevant to public health practice because the National Weather Service uses heat index for surveillance and for issuing heat warnings. This finding is also relevant given little previous work in comparing temperature metrics for ED visits for a specific outcome [85,86,87,88,89,90,91,92].

## 5. Conclusions

In evaluating associations between warm-season temperature and morbidity in Atlanta, this time-series study focused on a single outcome: ED visits for fluid and electrolyte imbalance. We assessed the effects of various temperature metrics and explored heterogeneity in these associations by potential determinants of heat susceptibility (sex, race, comorbid conditions, and measures of socioeconomic status). We found that warm-season temperature was associated with FEI for all temperature metrics considered and that risks were great for all populations, but that males experience somewhat greater susceptibility. We also observed heterogeneity in an unanticipated direction for underlying medical conditions, which may be related to competing diagnoses and requires further exploration. This work highlights the utility of FEI as an indicator of heat morbidity that affects all populations. It also highlights the health threat posed by warm-season temperatures, and raises considerations regarding assessment of susceptible populations in heat-health research. Specific results may be used to inform local heat-health warning systems, to improve estimations of the impact of heat on human health, and to highlight the dangers of dehydration in public health outreach on risks of heat exposure.

## Figures and Tables

**Table 1:** Descriptive statistics for various temperature metrics in Atlanta during the warm seasons (May–September), 1993–2012 (*n* = 3060 days, in units of °C). (Abbreviations: SD, standard deviation; Min, minimum; Pctl, percentile; Max, maximum).

Temperature Metric	Abbrev.	Mean	SD	Min	25th Pctl	50th Pctl	75th Pctl	Max
Maximum ambient air temperature	TMax	29.4	3.7	13.3	27.2	30.0	32.0	40.6
Minimum ambient air temperature	TMin	19.9	3.4	5.0	18.3	20.6	22.2	28.3
Average ambient air temperature	TAvg	24.7	3.4	11.7	22.8	25.3	27.0	33.9
Maximum apparent temperature	ATMax	30.3	3.9	14.2	28.0	30.8	33.0	40.2
Minimum apparent temperature	ATMin	21.4	4.1	5.1	19.3	22.6	24.4	30.4
Average apparent temperature	ATAvg	25.9	3.8	11.3	23.8	26.7	28.6	34.9
Maximum heat index	HIMax	30.6	4.6	13.2	27.8	30.8	33.8	43.4
Minimum heat index	HIMin	20.2	3.8	4.0	18.2	21.2	22.9	30.8
Diurnal change in air temperature	TDC	9.5	2.5	1.1	7.8	9.5	11.1	17.2
Diurnal change in apparent temperature	ATDC	8.8	2.2	1.4	7.5	8.8	10.3	17.2

**Table 2:** Spearman correlations between various temperature metrics in Atlanta during the warm seasons (May–September), 1993–2012 (*n* = 3060 days).

Temperature Metric	TMax	TMin	TAvg	ATMax	ATMin	ATAvg	HIMax	HIMin	TDC	ATDC
**TMax**	1.00									
**TMin**	0.75 *	1.00								
**TAvg**	0.95 *	0.91 *	1.00							
**ATMax**	0.96 *	0.84 *	0.97 *	1.00						
**ATMin**	0.71 *	0.99 *	0.89 *	0.82 *	1.00					
**ATAvg**	0.88 *	0.95 *	0.98 *	0.96 *	0.95 *	1.00				
**HIMax**	0.94 *	0.84 *	0.96 *	0.99 *	0.83 *	0.96 *	1.00			
**HIMin**	0.73 *	1.00 *	0.90 *	0.83 *	1.0 *	0.95 *	0.84 *	1.00		
**TDC**	0.43 *	−0.19 *	0.15 *	0.26 *	−0.23 *	0.03	0.23 *	−0.21 *	1.00	
**ATDC**	0.34 *	−0.27 *	0.07 *	0.22 *	−0.30 *	−0.04	0.20 *	−0.29 *	0.96 *	1.00

* *p* < 0.05.

**Table 3:** Estimated rate ratios (RR) for all fluid and electrolyte imbalance emergency department (ED) visits in relation to a change of approximately 1 IQR (from the 25th to 75th percentile) for various temperature metrics in Atlanta during the warm seasons (May–September), 1993–2012 (*n* = 3060 days).

Temperature Metric	Abbreviation	IQR (25th–75th Percentile, in °C)	RR (95% CI)
Maximum ambient air temperature	TMax	5 (27–32)	1.125 (1.102, 1.150)
Minimum ambient air temperature	TMin	4 (18–22)	1.105 (1.077, 1.133)
Average ambient air temperature	TAvg	4 (23–27)	1.134 (1.109, 1.159)
Maximum apparent temperature	ATMax	5 (28–33)	1.125 (1.103, 1.149)
Minimum apparent temperature	ATMin	5 (19–24)	1.080 (1.058, 1.102)
Average apparent temperature	ATAvg	5 (24–29)	1.137 (1.113, 1.162)
Maximum heat index	HIMax	6 (28–34)	1.122 (1.098, 1.146)
Minimum heat index	HIMin	5 (18–23)	1.096 (1.072, 1.120)
Diurnal change in air temperature	TDC	3 (8–11)	1.077 (1.058, 1.096)
Diurnal change in apparent temperature	ATDC	3 (7–10)	1.055 (1.036, 1.073)

**Table 4:** Descriptive statistics of fluid and electrolyte imbalance ED visits stratified by modifiers of interest in Atlanta during warm seasons (May–September), 1993–2012.

Modifier	Strata	# of Days	Total FEI ED Visits	Total FEI ED Visits with Modifier Information ^1^	Mean (SD) of Daily FEI ED Visits
All FEI ED visits	---	3060	66,369	---	21.7 (12.8)
Sex	Female	3060	38,426	65,713	12.6 (7.6)
	Male	3060	27,287		8.9 (6.1)
Race ^2^	Black	2601	14,972	44,384	5.8 (5.3)
	Hispanic	2601	1938		0.7 (1.2)
	Other	2601	1646		0.6 (0.9)
	White	2601	25,828		9.9 (6.6)
Comorbid Congestive Heart Failure	Present	3060	3566	66,369	1.2 (1.4)
	Absent	3060	62,803		20.5 (12.0)
Comorbid Renal Disease	Present	3060	9477	66,369	3.1 (3.6)
	Absent	3060	56,892		18.6 (10.1)
Comorbid Diabetes	Present	3060	9737	66,369	3.2 (3.1)
	Absent	3060	56,632		18.5 (10.4)
Poverty Area ^3^	Yes	3060	11,623	66,343	3.8 (3.5)
	No	3060	54,720		17.9 (10.2)
Undereducated Area ^4^	Yes	3060	9005	66,343	2.9 (2.0)
	No	3060	57,338		18.7 (12.0)

^1^ There were 656 visits missing sex data, 21,985 visits missing race data, 26 visits missing poverty area data, and 26 visits missing undereducated area data; ^2^ The number of days with race data available was lower than 3060 as race information was not available for the 2007–2009 period; ^3^ “poverty area” defined as Zip Code Tabulation Areas (ZCTAs) with ≥20% of the population living below the federal poverty line; ^4^ “undereducated area” defined as ZCTAs with ≥25% of the adult population without a high school graduation.

**Table 5:** Estimated rate ratios for fluid and electrolyte imbalance ED visits in relation to a change of approximately 1 IQR (from the 25th to 75th percentile) for TMax (27 °C to 32 °C) and ATMax (28 °C to 33 °C) in Atlanta during the warm seasons (May–September), 1993–2012 (*n* = 3060 days) and 1998–2012 (*n* = 2295 days).

Modifier	Strata	TMax RR (95% CI) All Years Included (1993–2012)	TMax RR (95% CI) Reduced Period (1998–2012)	ATMax RR (95% CI) All Years Included (1993–2012)	ATMax RR (95% CI) Reduced Period (1998–2012)
Overall		1.125 (1.102, 1.150) **	1.121 (1.096 1.1460) **	1.125 (1.103, 1.149) **	1.123 (1.099, 1.147) **
Sex	Female	1.088 (1.060, 1.118) **	1.085 (1.054, 1.117) **	1.085 (1.057, 1.114) **	1.085 (1.055, 1.116) **
	Male	1.176 (1.138, 1.216) **^,^^	1.169 (1.128, 1.210) **^,^^	1.181 (1.143, 1.219) **^,^^	1.174 (1.135, 1.214) **^,^^
Race ^1^	White	1.141 (1.102, 1.180) **	1.128 (1.087, 1.170) **	1.127 (1.090, 1.164) **	1.116 (1.077, 1.156) **
	Nonwhite	1.157 (1.113, 1.202) **	1.145 (1.099, 1.193) **	1.159 (1.116, 1.204) **	1.150 (1.105, 1.197) **
	Black	1.143 (1.095, 1.193) **	1.132 (1.081, 1.185) **	1.143 (1.097, 1.192) **	1.135 (1.086, 1.187) **
	Hispanic	1.366 (1.211, 1.541) **	1.368 (1.209, 1.549) **	1.304 (1.159, 1.466) **	1.304 (1.155, 1.471) **
	Other	--	1.028 (0.902, 1.171)	--	1.072 (0.962, 1.195)
Comorbid Congestive Heart Failure	Present	--	1.048 (0.959, 1.145)	--	1.069 (0.981, 1.166)
	Absent	1.130 (1.106, 1.155) **	1.125 (1.099, 1.151) **	1.129 (1.105, 1.153) **	1.126 (1.101, 1.151) **
Comorbid Renal Disease	Present	1.048 (0.995, 1.104)	1.049 (0.993, 1.108)	1.069 (1.016, 1.125)	1.071 (1.014, 1.130) *
	Absent	1.137 (1.112, 1.164) **^,^^	1.137 (1.112, 1.164) **^,^^	1.134 (1.110, 1.159) **^,^^	1.132 (1.106, 1.159) **
Comorbid Diabetes	Present	1.096 (1.040, 1.156) *	1.100 (1.040, 1.163) *	1.102 (1.047, 1.160) *	1.106 (1.047, 1.167) *
	Absent	1.130 (1.105, 1.156) **	1.124 (1.098, 1.152) **	1.129 (1.105, 1.154) **	1.126 (1.100, 1.152) **
Poverty Area	Yes	1.132 (1.079, 1.187) **	1.130 (1.075, 1.188) **	1.131 (1.080, 1.185) **	1.128 (1.074, 1.183) **
	No	1.125 (1.099, 1.151) **	1.119 (1.092, 1.147) **	1.125 (1.100, 1.150) **	1.122 (1.096, 1.149) **
Undereducated Area	Yes	1.175 (1.114, 1.239) **	1.172 (1.104, 1.244) **	1.183 (1.124, 1.245) **	1.179 (1.113, 1.248) **
	No	1.118 (1.093, 1.144) **	1.114 (1.087, 1.141) **	1.116 (1.092, 1.141) **	1.115 (1.089, 1.141) **

** *p* < 0.0001; * *p* < 0.05; ^ test for heterogeneity *p*-value < 0.05; ^1^ The number of days with race data available was lower than 3060 as race information was not available/was not acquired for the 2007–2009 period.
